# Mucopolysaccharidosis Type VI, an Updated Overview of the Disease

**DOI:** 10.3390/ijms222413456

**Published:** 2021-12-15

**Authors:** Francesca D’Avanzo, Alessandra Zanetti, Concetta De Filippis, Rosella Tomanin

**Affiliations:** 1 Laboratory of Diagnosis and Therapy of Lysosomal Disorders, Department of Women’s and Children’s Health, University of Padova, 35128 Padova, Italy; francesca.davanzo@unipd.it (F.D.); alessandra.zanetti@unipd.it (A.Z.); concetta.defilippis@studenti.unipd.it (C.D.F.); 2 Fondazione Istituto di Ricerca Pediatrica Città della Speranza, Corso Stati Uniti 4, 35127 Padova, Italy

**Keywords:** lysosomal storage disorder, mucopolysaccharidosis type VI, Maroteaux–Lamy syndrome, dermatan sulfate, chondroitin 4-sulfate, *ARSB*, ASB, N-acetylgalactosamine 4-sulfatase, enzyme replacement therapy

## Abstract

Mucopolysaccharidosis type VI, or Maroteaux–Lamy syndrome, is a rare, autosomal recessive genetic disease, mainly affecting the pediatric age group. The disease is due to pathogenic variants of the *ARSB* gene, coding for the lysosomal hydrolase N-acetylgalactosamine 4-sulfatase (arylsulfatase B, ASB). The enzyme deficit causes a pathological accumulation of the undegraded glycosaminoglycans dermatan-sulphate and chondroitin-sulphate, natural substrates of ASB activity. Intracellular and extracellular deposits progressively take to a pathological scenario, often severe, involving most organ-systems and generally starting from the osteoarticular apparatus. Neurocognitive and behavioral abilities, commonly described as maintained, have been actually investigated by few studies. The disease, first described in 1963, has a reported prevalence between 0.36 and 1.3 per 100,000 live births across the continents. With this paper, we wish to contribute an updated overview of the disease from the clinical, diagnostic, and therapeutic sides. The numerous in vitro and in vivo preclinical studies conducted in the last 10–15 years to dissect the disease pathogenesis, the efficacy of the available therapeutic treatment (enzyme replacement therapy), as well as new therapies under study are here described. This review also highlights the need to identify new disease biomarkers, potentially speeding up the diagnostic process and the monitoring of therapeutic efficacy.

## 1. Introduction

Mucopolysaccharidosis type VI (MPS VI), or Maroteaux–Lamy syndrome (MIM# 253200), is a rare, inherited, autosomal recessive metabolic disorder caused by low to absent activity of the lysosomal enzyme N-acetylgalactosamine 4-sulfatase (arylsulfatase B; ASB; EC 3.1.6.12) that catalyzes one of the steps of degradation of the glycosaminoglycans (GAGs) dermatan sulfate (DS) and chondroitin 4-sulfate (CS). This leads to a progressive accumulation of these molecules into lysosomes and extracellular matrix, with consequent cell and tissue injury that progressively determines a series of multi-systems/organs failure, taking to severe clinical manifestations [1].

Pierre Maroteaux and Maurice Lamy first reported MPS VI in 1963, describing it as a novel dysostosis with increased urinary excretion of chondroitin sulfate [2].

After noticing the deficit in patients’ fibroblasts, Baron & Neufeld first described the biochemical deficit specifically related to the Maroteaux–Lamy syndrome in 1972. They specified that the so-called “corrective factor” was deficient in a group of siblings affected by the disease [3]. From an historical point of view, this was the fifth corrective factor suspected at the base of the different syndromes belonging to the mucopolysaccharidoses group, following factors causing Hunter syndrome, Hurler–Scheie syndrome and two “biochemical subclasses” of Sanfilippo Syndrome [3].

In this review we present an updated overview of the MPS VI from the clinical, diagnostic, and therapeutic sides. To this aim, a literature search was performed in PubMed and Google using the search terms “mucopolysaccharidosis type VI”, “MPS VI”, “Maroteaux–Lamy syndrome” associated with specific terms related to the different issues reviewed (last literature search: 31 August 2021). Then, retrieved publications were filtered, so as to focus on the more recent papers that were not included in the last review by Harmatz and Shediac [1].

## 2. Epidemiology

The prevalence of MPS VI is quite variable among the different populations and it is estimated to range from 0.0132 per 100,000 live births in Poland [4] to 7.85 per 100,000 live births in Eastern Saudi Arabia [5]. However, in specific small populations, high rates of endogamy and parental consanguinity could determine an increase of the prevalence up to 20 per 100,000 live births as reported by Costa-Motta in a small town in Northeast Brazil [6]. When considering all MPSs, MPS VI is the most frequent in Saudi Arabia (46% of all MPS cases) and in Malaysia (40%), while the lowest frequencies are reported for Poland (1%) and South Korea (1.4%) [7]. However, these are likely underestimated prevalences, as the diagnoses rely on the clinical detection of specific signs and symptoms. A more accurate estimation of the prevalence of this disorder in the different countries would be likely obtained by the precocious identification of MPS VI patients through the application of newborn screening programs [7], at the moment still in their pilot phase [8,9,10].

## 3. Molecular Basis

**Figure 1:**
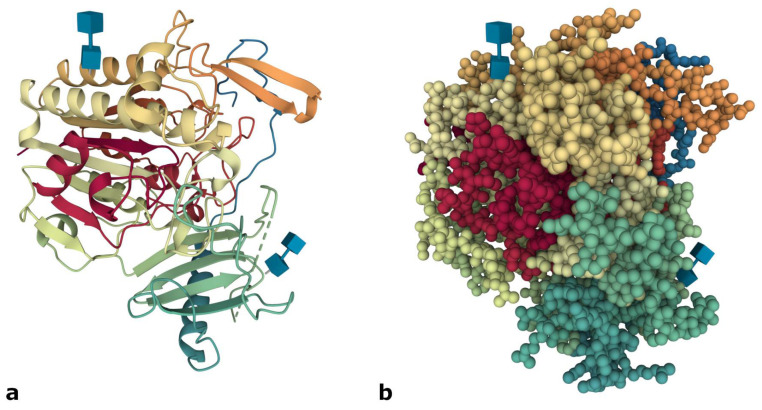
Crystal structure of ASB protein shown by cartoon (**a**) and spacefill (**b**) representations. Image from the RCSB PDB (http://www.rcsb.org/, accessed on 3 September 2021) [15] of PDB ID 1FSU [12], created with Mol* viewer [16].

The *ARSB* gene (Ensembl ID: ENSG00000113273) maps on chromosome 5q13-14 and spans a region of approximately 208 kb; it contains eight exons and encodes a transcript of 4852 bp (NM_000046.5), which is processed and translated into the 533 amino acids- mature form of the ASB protein (Ensembl release 104, May 2021) [11]. According to the crystallographic structure, the ASB enzyme is a monomeric protein composed by a larger N-terminal domain and a smaller C-terminal one [12] (Figure 1). The active site pocket is located in the N-terminal domain and is composed of 10 conserved amino acid residues, including the Cys91, which is post-translationally modified to 3-oxoalanine (α-formylglycine) [13], a residue necessary for the catalytic activity of type I sulfatases [14].

**Figure 2:**
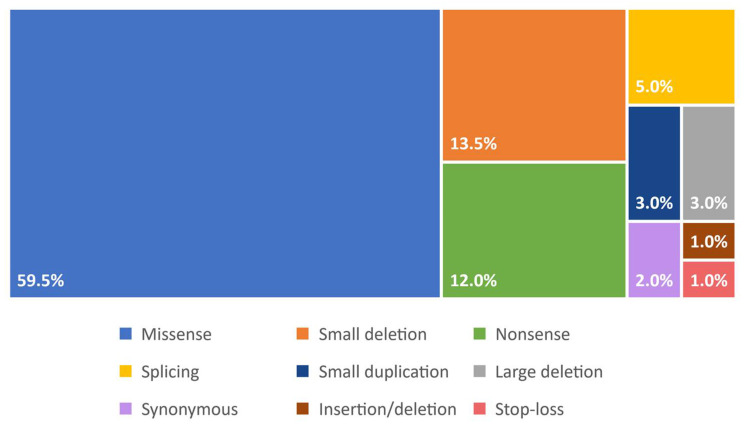
Distribution of variant types in the *ARSB* gene. Data from [17] with permission.

Up to date, more than 220 unique variants have been reported for the *ARSB* gene: most of them are missense variants (59.5%); followed by small deletions (13.5%); nonsense (12.0%); splice site and intronic variants (5.0%); small duplications (3.0%); and large deletions (3.0%) [17] (Figure 2). The gene presents a high genetic heterogeneity with almost one-third (31.7%) of unique variants reported only once and an additional 28.5% reported twice [17]. The alleles c.454C > T [p.(Arg152Trp)] and c.962T > C [p.(Leu321Pro)] are the most common variants and are diffused predominantly in Russia and Turkey respectively. Although more than half of MPS VI patients reported in literature are homozygotes, genotype-phenotype correlation is not so straightforward, with only a few alleles for which a correlation with the phenotype can be assessed for most of the reported cases [1,17].

Since the 2018 *ARSB* mutation update [17], different groups, mainly from the Middle East area [18,19,20,21,22,23,24,25,26,27,28,29], have published several papers describing *ARSB* gene variants. Considering the small percentage of splicing variants identified up to date in *ARSB* gene with respect to the reported frequency of splicing variants in human disease [30,31], Broeders and colleagues have lately applied to *ARSB* gene a general method for the unbiased identification and quantification of mRNA [32]. This allowed the identification of novel aberrant transcripts in four patients with three different genotypes, of 12 MPS VI patients included in the analysis [32]. Consistently with these findings, a deep intronic variant [c.1142 + 581A > G] in a homozygosis status has been recently identified by whole genome sequencing in two affected siblings who had consanguineous parents [33].

## 4. Clinical Features and Degrees of Severity

**Figure 3:**
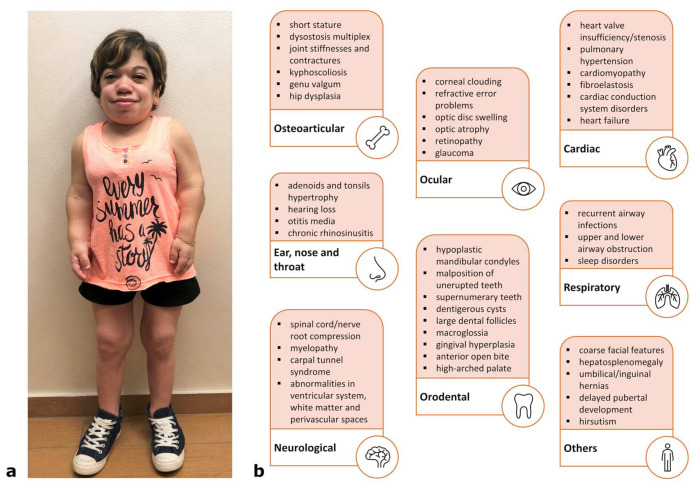
(**a**) A 20-year-old female MPS VI patient, showing facial dysmorphism, short stature and claw hands; (**b**) main clinical manifestations associated with the disease.

The classical clinical features of Maroteaux–Lamy syndrome are represented by an important impairment of the osteoarticular system, with dysostosis multiplex, short stature and motor dysfunction [34], among others (Figure 3a). In addition, ocular (above all corneal clouding) and ENT (ear, nose, throat) manifestations were registered quite commonly [1], as well as orodental anomalies [35,36]. These last are particularly noticeable in MPS VI, presenting in most patients with hypoplastic condyles, malposition of unerupted teeth, large dental follicles and open bite [35]. Finally, other signs, such as organomegaly and cardio-respiratory insufficiencies, typical of MPSs in general, are also present in MPS VI [1]. Figure 3b summarizes the clinical manifestations mainly reported in the patients.

In relation to nervous system involvement, some brain structural abnormalities, including white matter lesions, perivascular spaces, communicating hydrocephalus and ventricular enlargement, have been described, together with spinal cord compression, myelopathy and carpal tunnel syndrome [34,37].

Although most of the literature reports rare neurocognitive and behavioral abnormalities in these patients, more recently, the absence of studies robustly addressing these aspects has been underlined as an important issue [37]. Such studies would help a more correct diagnosis and prognosis on patients’ neurological development. They may also allow attention to be focused on all neurocognitive aspects, including intelligence, attention, memory functions and behavior. In particular, low IQ scores were detected in some severe MPS VI patients, independently from their familiar and social background factors [38]. Furthermore, attention skills were shown to be significantly impaired in attenuated MPS VI patients and negatively associated with somatic disease progression [39].

In the past, three (severe, intermediate, and mild) [40] or two (severe and mild) [41] classes of phenotypes were used to describe MPS VI clinical features, on the basis of the disease severity. Today, a continuum of phenotypes is commonly recognized, from a rapidly progressing form to a slowly progressing one, based on the age at onset and, as just stated, on the rate of progression and severity of clinical signs and symptoms [1].

### 4.1. Rapidly Progressing Forms

Rapidly progressing forms present earlier—before the age of 2—with typical osteo-articular signs taking, after the first year of life, to a rapidly decreasing percentile of the height, with respect to the Centre for Disease Control standard curves (https://www.cdc.gov/, accessed on 3 September 2021), and falling below the fifth percentile at around 3 years of age [42].

It is now commonly accepted that severe patients are defined based on a rapid progression of the clinical signs, mainly osteoarticular ones, and on their height that, in these patients, hardly exceeds 120 cm [43,44]. Severe patients usually come to clinical attention due to problems with their skeleton, joints or both; thus, their claw hands might be the first sign observed by orthopedists, though they typically also present with a marked facial dimorphism, which can be easily observed at physical examination. Typical osteo-articular signs also include kyphosis, scoliosis, pectus carinatum and, in the course of the disease, patients suffer from difficult mobility, due to leg and hip problems, which might force some of them to the wheelchair [45].

In these patients, corneal clouding, first described in 1971 by Stevenson [46], very often compromises vision and is sometimes treated by corneal transplant [47]. The sign, present in most Maroteaux–Lamy patients, has revealed to be quite useful in the diagnosis of the patients, helping to address a possible differential diagnosis. In addition, glaucoma and optic nerve abnormalities have been often reported for MPS VI [48]. 

As in the severe forms of most MPSs, in these subjects an important respiratory failure can progressively develop from the childhood [44]. This is often accompanied by significant valvulopathies, involving mainly mitral and tricuspid but also the aortic valves, which often require surgical interventions for valve replacement. Also, severe heart failure represents a common finding in MPS VI patients and is the major cause of death in severe subjects, between the second and the third decade of life [44].

Absent or delayed puberty has also been described often in these patients [44,49].

Biochemically, the rapidly progressing forms might present at diagnosis with more elevated levels of urinary GAGs [43,50], than those commonly measured in slowly progressing forms.

### 4.2. Slowly Progressing Forms

Attenuated forms of MPS VI, or slowly progressing forms, are associated with a slower clinical course and, generally, they present with less pronounced symptoms or even with atypical ones. Sometimes fewer organ-systems are affected [1] and often these patients present with normal or only mildly coarsened facial features, thus lacking one of the first signs helping to start the diagnostic process. This renders them more difficult to diagnose and in rare cases they happen to be correctly diagnosed after several years of misdiagnosis, or even they remain unreported; according to data so far reported in the literature [20,51], the age at diagnosis ranges from 9 to 42 years with an average of 23.5 years. Most attenuated MPS VI patients described so far in the literature initially present with osteoarticular symptoms (joint stiffness and pain), at a mean age of 10 years, but also in adult age. These generally progress to more serious symptoms, again involving the osteoarticular system (joint degeneration, carpal tunnel syndrome, hip disease), but also other districts (sleep apnea, reduced pulmonary function, valvulopathies) [20,51]. An index case, representing the “attenuated end of the clinical disease spectrum” was described by Brooks and colleagues in 2005 in a Dutch patient presenting no clinical signs of the disease and a residual ASB activity corresponding to 5% [52]. However, the paper did not report the causes taking to the medical attention [52]. Other significant slowly progressing MPS VI phenotypes have been described along the years. Scarpa and colleagues reported in 2010 [53] the case of two sisters who had remained misdiagnosed for 35 and 38 years respectively, although a careful examination would have identified in both subjects some clinical signs closely suggesting a lysosomal storage disorder. They were taller than 140 cm, and one of them was pregnant of her third child, while the other two were born through a caesarean section. The two sisters were compound heterozygous for the variant c.629A > G [p.(Tyr210Cys)], just afterwards associated to an attenuated clinical phenotype in a homozygote patient [54], and for the variant c.904G > A [p.(Gly302Arg)], whose pathogenic meaning is still of uncertain significance [17]. 

In 2012, Thümler and colleagues described nine patients with attenuated phenotypes. Although all classified as presenting an attenuated form, they showed involvement of different organs, with common features being a slightly reduced height and a cardiac involvement [55]. Moreover, seven out of nine patients presented with corneal clouding at enrollment in the study, but this did not affect their visual acuity [55], while hearing problems were only registered in one patient. Since visual and auditory problems had been commonly reported before for MPS VI patients [47,56], Thümler and colleagues concluded that these features are not predominant findings of MPS VI patients suffering from the slowly progressing form of the disease [55].

## 5. Diagnosis

The diagnostic iter of MPS VI commonly starts with the identification of specific clinical signs, suggestive of a metabolic disorder, which are generally referred to a metabolic specialist or a clinical geneticist [1]. The main indicative signs might be one or more of the following: short stature; bone-related dysostosis multiplex; hepatomegaly, splenomegaly, or both; macrocephaly; inguinal or umbilical hernia; corneal clouding; cardiac valve thickening [44]. However, although extremely rare, very mild patients with no obvious clinical signs of MPS VI have been reported in the literature [52].

As next steps, a quantitative GAG analysis by DMB-based assay, demonstrating an increase of total urinary GAGs or a qualitative analysis revealing an increase of dermatan sulphate (DS) are performed. Regarding qualitative GAG analysis, in the last 10 years more specific methods based on LC-MS/MS have been implemented also in diagnostic settings both for body fluids and for dried blood spots [57]. GAG analysis is generally followed by enzymatic assay evaluating ASB enzymatic activity on leucocytes or fibroblasts [58]. These assays should include the evaluations of one or more additional sulphatases, so as to rule out the diagnosis of multiple sulfatase deficiency (MSD), as well as the assay of a mannose-6-phosphate guided reference enzyme to exclude mucolipidosis type II alpha/beta (I-cell disease), only in case of enzymatic assay performed in fibroblasts [59].

An independent confirmation of MPS VI diagnosis is obtained by molecular genetic testing of *ARSB* gene, which allows the detection of the variants associated with the disease. Generally, molecular analysis is performed on both parents so as to verify whether the variants are located on opposite chromosomes (segregation analysis) and, in case of only one variant detected, to confirm the status of homozygosis or, alternatively, to evidence the presence of large deletions [59]. In any case the results of the molecular analysis in the proband and in both parents is useful to support the genetic counseling, especially in case of preconception or prenatal testing, in families with MPS VI patients [17].

Differential diagnosis should include MPS I, MPS II, MPS IVA, MPS VII and multiple sulfatase deficiency (MSD). It should also include sialidosis, mucolipidosis (ML) type II alpha/beta, ML III alpha/beta, ML III gamma and ML IV [44].

Prenatal testing is generally performed evaluating ASB activity of fresh or cultured chorionic villus cells obtained by villocentesis or of cultured amniotic fluid cells obtained via amniocentesis. If an index case is present in the family and his or her genotype is known, molecular genetic testing could be also performed to support/confirm the results of the enzymatic assay, thus substantially increasing the reliability of the prenatal diagnosis [44,58].

### Newborn Screening (NBS)

NBS for MPS VI has been developed only in a few countries. In Taiwan, this is part of a large-scale national newborn screening program of lysosomal storage disorders based on the MS/MS technology. The analysis of 130,175 newborns evidenced two positive neonates, but these were not confirmed by leukocytes activity and urinary GAG analysis [8]. Moreover, Chien and colleagues developed an 8-plex assay through which they evaluated more than 70,000 neonates leading to the detection of no positive cases [9]. In USA, a pilot study for five different MPSs including MPS VI, performed in over 106,000 newborns in Washington State, evidenced four positive neonates who, however, were not confirmed by a second-tier analysis [10].

## 6. Treatment

### 6.1. Management of Symptoms

Given the involvement of multiple organs and systems, MPS VI patients need a continuous multi-disciplinary management approach to monitor all the disease manifestations [1]. Recently, evidence- and consensus-based recommendations for the management of patients with MPS VI were published [60]. These guidelines evidenced the recommended routine monitoring and assessments that should be used to follow the natural history of MPS VI patients, and the interventions to manage the common symptoms. The recommended assessments included the following examinations: physical examination; radiology; endurance; growth; urinary glycosaminoglycan levels; cardiac function; neurological examination; upper limb function; respiratory function and sleep disorder; ear-nose-throat (ENT); ophthalmological function; evaluation of oral health; disease burden; and physical therapy [60]. Moreover, subjects affected by MPS VI may need surgical interventions during their life such as corneal transplant, cardiac valve replacement and cervical decompression, together with oro-dental surgeries necessary to reduce eating and chewing difficulties of the patient. In all these cases, as well as in other types of investigation, patients may likely need anaesthetic procedures; these are highly risky for MPS VI patients, due to potential airway difficulties with mask ventilation or endotracheal intubation (or both) and need to be carefully evaluated as regarding risks vs. benefits [61]. Once these procedures are applied, patients must be strictly monitored and supervised by experienced anaesthetists [60].

### 6.2. Enzyme Replacement Therapy (ERT)

Enzyme replacement therapy is based on the weekly intravenous infusion with the recombinant form of human ASB or galsulfase (Naglazyme^®^, BioMarin Pharmaceutical Inc., Novato, CA, USA) and to date is the only specific treatment developed for MPS VI [62]. Feasibility of enzyme replacement therapy for MPS VI was first evaluated in vitro: the recombinant N-acetylgalactosamine 4-sulfatase enzyme obtained in CHO-DKI cells was demonstrated to be similar to the endogenous enzyme, to be able to correct the enzyme defect and consequently to reduce GAG storage in cultured MPS VI fibroblasts [63]. These results opened the way to preclinical studies performed in the feline model of MPS VI [64,65], first with the human recombinant enzyme and then with the feline form, showing the efficacy of this treatment to alter the course of the disease [66,67,68,69]. Cats treated from birth to about 170 days of age evidenced a significant decrease in total urinary GAG content, skeletal improvement and reduction of lysosomal storage in all tissues examined, with the exception of cartilage, cornea, and white blood cells [68]. Moreover, a safety profile and a more pronounced overall improvement in the disease condition were evidenced in cats treated from birth, compared to cats treated at a later age [70].

The following clinical studies confirmed safety and efficacy in humans leading to the first approval by USA in 2005, followed by Europe in 2006, and then by other countries [71,72,73]. The phase I/II, randomized, two-dose, double-blind clinical study evidenced in six patients with variable severity a rapid reduction in urinary GAG levels, after 24–48 weeks of treatment. Moreover, an improved endurance was observed, more marked in patients receiving the higher dose (1.0 mg/kg) with respect to the lower one (0.2 mg/kg) [71]. A subsequent phase II, open-label study in 10 patients with rapidly advancing disease was performed using the 1.0 mg/kg dose; after 48 weeks of treatment reduction of urinary GAGs, improvement of endurance and of joint mobility were observed, as well as a positive safety outcome of the treatment [72]. Finally, a phase III randomized, double-blind, placebo-controlled, multicenter study evaluated 39 patients treated for 24 weeks, then extended to 48, further confirming efficacy and safety results of the previous phase II trial [73].

Continuation of the three clinical studies for a longer period (97–260 weeks) was allowed to further confirm that weekly administration of 1.0 mg/kg of galsulfase determined an improvement in endurance as well as a reduction in urinary GAG levels, also showing that the safety profile of the treatment remained sufficient [74].

Further observational data on ERT clinical efficacy on a larger population and over a long period of time is still being collected by the MPS VI Clinical Surveillance Program (CSP), a voluntary, multinational, multicentre disease registry established in 2005 by BioMarin to address a post-marketing commitment assigned by the FDA and the EMA [56]. Data collected from CSP further confirmed positive impact of ERT on several manifestations of MPS VI, including endurance, liver and spleen size, and pulmonary function [56].

In the last 10 years, increasing available data on ERT efficacy has led to the publication of the results of several long-term studies. The first, published in 2014 by Giugliani et al. [50], was a cross-sectional survey study in 121 MPS VI patients treated for an average of 7 years: collected data evidenced improvements of pulmonary function, endurance, a stabilized cardiac function, and an increased survival. Another study by Horovitz evidenced, in 32 patients treated for about 10 years, that starting ERT before 5 years of age has positive effects on growth velocity, major respiratory complications and mortality rate [75]. Four infants (aged 3.3–12.7 months) after 1 year treatment showed a reduction in urinary GAGs and a normal growth during the first year of treatment, but a progression of skeletal manifestations [76]. Three out of these four children presented, after 7.7–9.8 years of treatment, a slowdown of the clinical course of the disease with exception of skeletal and eye disease that appeared to progress [77].

Furthermore, clinical observations on siblings have demonstrated that an early ERT treatment can be effective in preventing and slowing down disease progression, but with still limited effect on skeletal symptoms and corneal clouding [78,79,80,81]. Moreover, early initiation of ERT has positive effects on children growth with an age- and severity-dependent impact: younger and more severe children (high urinary GAG excretion patients) seem to receive more benefit [49,50,82].

The effect of ERT on adult patients was evaluated from CSP data of 51 patients who started ERT as adults (≥16 years) and had received galsulfase for ≥6 months, evidencing that endurance and pulmonary function remained stable over a median treatment of 7 years, while cardiac disease still progressed [83].

Evaluation of ERT outcomes at either end of the disease spectrum, i.e., patients with pretreatment urinary GAG levels <100 μg/mg and ≥200 μg/mg creatinine, was also performed using data from CSP. Both groups evidenced a stabilized endurance and pulmonary function after 6–8 years of follow-up; however, no effects were registered on cardiac valve disease [84].

Finally, a systematic review [85] of the published observational studies, evaluating the efficacy of ERT for MPS VI, was recently performed. In the 18 included studies, 7 out of the 14 selected clinical outcomes, including patient survival, quality of life, respiratory function, joint mobility, physical resistance, reduction of urinary glycosaminoglycans and growth, showed positive results. The remaining outcomes resulted stable (hearing function and cognitive development) or presented inconclusive results (cardiac function, visual acuity, sleep apnea, and liver and spleen size).

#### Intra-Articular ERT

As articular cartilage, due to its avascular nature, is resistant to ERT, injection of enzyme directly into the joint has been evaluated in MPS VI cats with encouraging results [86,87]. The following small-scale clinical trial, performed in two patients, evaluated the feasibility of such an approach and partially confirmed the preclinical encouraging data, with mild improvement of the hip joint or at least a lack of articular deterioration [88]; however, the necessary further large-scale studies are still missing.

### 6.3. Bone Marrow Transplantation (BMT) and Hematopoietic Stem Cells Transplantation (HSCT)

The transplantation of multipotent hematopoietic stem cells, derived from bone marrow, peripheral blood, or umbilical cord blood of a healthy donor is a possible therapeutic option for MPSs. It was successfully applied mainly to MPS I patients, where it was able to partially treat CNS involvement. As for MPS VI, an incidence of 36% of graft-versus-host disease at 100 days post transplantation and a survival of 66% after 1 year were observed in a retrospective study of 45 patients [89]. The considerable associated risks of graft failure, graft-versus-host disease, infection during immune suppression and endocrine and gonadal failure, as well as the inability to correct or prevent musculoskeletal symptoms and corneal clouding, currently reduce HSCT application to MPS VI [1]. However, the combined treatment of ERT and HSCT, able to reduce HSCT morbidity and mortality in MPS I, gave good results also in a 3-year-old girl with MPS VI. An improvement in respiratory function, hepatosplenomegaly and joint range of motion was observed, although the progression of musculoskeletal complications and cardiac valve disease was not prevented [90]. The administration of ERT, started 10 years after a successful bone marrow transplantation, also improved the joint range of motion of multiple joints and the outcome of walking endurance tests [91].

### 6.4. Other Therapies

Because of the very limited ability of ERT and HCST to correct pathological abnormalities in cardiac valves, bone, cartilage, eye and CNS in MPS VI, many other therapeutic strategies are being developed, in some cases as combination with the available therapies. They include alternative administration routes for ERT, gene therapy and substrate reduction therapy approaches, as well as anti-inflammatory and pharmacological read-through drugs (Table 1).

#### 6.4.1. Gene Therapy

The most studied gene therapy strategy for MPS VI exploits adeno-associated viral vectors. AAV-2-mediated subretinal delivery of *ARSB* gene corrected the disease phenotype in the retinal pigment epithelium of MPS VI cat model [94]. In the same model, the intravenous (i.v.) administration of AAV2/8 vector carrying the *ARSB* gene, under a liver-specific promoter, induced an *ARSB* expression up to 1 year. This produced clearance of GAG storage, reduction of heart valve thickness, improvement of long bone length and spontaneous mobility [95], in absence of pre-existing immunity to AAV8 [96]. In the MPS VI mouse model, a single i.v. administration of this vector produced at least the same efficacy (in reducing GAG level in urine and tissues, and in improving skeletal abnormalities and motor performances) of a weekly administration of ASB enzyme conducted for 1 year. Moreover, the gene therapy approach produced increased and stable levels of circulating enzyme for 1 year, thus allowing the single administration, while ERT presented typical peak-and-drop serum kinetics [98].

Although AAV vectors are widely used for in vivo gene therapy protocols, safety concerns have been raised due to some events of insertional mutagenesis and the subsequent development of hepatocellular carcinoma (HCC) observed in newborn mice treated with high doses of AAV [109]. As for AAV-treated young adult MPS VI mice, HCCs developed in 6 out of 76 mice, with only one occurrence of pathological integration; no evidence of liver tumorigenesis was found in juvenile AAV-treated cats up to 8 years after vector administration [97]. In terms of general health, haematology, clinical chemistry and histopathology, no toxicity was observed in mice, except for a transient increase in alanine aminotransferase in females, and thyroid epithelial hypertrophy [99].

A combinatorial gene therapy/ERT approach was tested in MPS VI mouse model for 6–7 months, with the aim of reducing the risks of genotoxicity associated to high dose of gene therapy as well as ameliorating biodistribution, quality of life, immune response and high costs associated with ERT. This strategy, consisting in low doses of AAV2/8 vector and a monthly scheduled ERT (less frequent than canonical ERT administration), obtained the same efficacy in terms of reduction of urinary GAGs and storage in myocardium and heart valves, compared with the single therapies [110].

Besides adeno-associated viral vectors, retroviral and lentiviral vectors were also tested for MPS VI therapy. Retroviral vectors have proven to be able to induce high levels of *ARSB* expression and a reduction of GAG storages in primary cultures of cat muscle cells [92]. Moreover, MPS VI cats i.v.-injected with gamma retroviruses expressing feline *ARSB* presented with higher body weights, longer appendicular skeleton lengths, reduced articular cartilage erosion and reduced aortic valve thickening and aortic dilation compared with untreated MPS VI cats. However, some aspects of bone disease, such as cervical vertebrae bone lengths, remained difficult to treat [93].

HIV-1-based lentiviral vectors expressing feline 4-sulphatase were used to transduce human MPS VI fibroblasts and feline MPS VI chondrocytes. A long-lasting enzyme expression, reaching at least 41 days in fibroblasts, was obtained, together with a dose-dependent reduction of GAG storage in both cell types. The same construct, after injection into rat knee joints, produced a widespread expression of the marker gene in the synovial membrane and the fascia covering the cruciate ligaments and meniscus, for at least 8 weeks after injection; no transduction of chondrocytes or ligament cells was observed [100].

Lentiviral vectors coding for rat 4-sulfatase also proved to efficiently transduce human hematopoietic stem cells (HSCs) and mesenchymal stem cells (MSCs) derived from bone marrow and from dental pulp, resulting in a high induced enzyme activity both in the cell layers and in their media [101].

#### 6.4.2. Substrate Reduction Therapy

As a substrate reduction therapy, aiming to reduce GAG accumulation by interfering with their biosynthetic pathway, an orally administrable small molecule called Odiparcil (Inventiva Pharma, Daix, France) was developed for MPS VI. Odiparcil works as decoy substrate for the β-1,4-galactosyltransferase (B4GalT7) enzyme, required for the synthesis of O-glycosylated proteoglycans. It diverts the synthesis of cellular glycosaminoglycans into secreted soluble species, mainly CS-GAGs, thus reducing intracellular accumulation. Odiparcil has proven to reduce intracellular CS in human MPS VI fibroblasts, and to ameliorate tissue GAG accumulation, corneal morphology and opacification and cartilage thickening in MPS VI mice after oral treatment [102,103].

A phase II clinical trial study (EudraCT number: 2017-002158-35; ClinicalTrials.gov Identifier: NCT03370653) in MPS VI patients older than 16 years was completed in 2019. A good safety profile and some improvements in pulmonary functions and corneal clouding were described; however, due to the small number of subjects, further investigations are needed to confirm the efficacy of this strategy in MPS VI patients.

#### 6.4.3. Anti-Inflammatory Drugs

Since inflammation was proven to be important in MPS pathology, including skeletal involvement [104], the effect of combining anti-inflammatory drugs with ERT was examined in MPS VI models.

Infliximab, a chimeric monoclonal antibody against human TNF-α, when injected into MPS VI rats prevented some inflammatory responses in chondrocytes and fibroblast-like synoviocytes, reduced the apoptosis in articular chondrocytes and the infiltration of synovial tissue into the underlying bone [104]. After 8 months of treatment in combination with ERT, a reduction of TNF-alpha was observed in articular cartilage, together with a restored collagen IIA1 expression and a reduction of apoptosis in chondrocytes. Tracheal deformities and ceramide levels in trachea were reduced; motor activity and mobility, improved by ERT, were also significantly enhanced by the combination therapy [111].

Pentosan polysulfate, an oral medication with anti-inflammatory and pro-chondrogenic properties was shown to reduce anti-inflammatory markers in serum, in tissues and in cultured articular chondrocytes of MPS VI rats. In addition, improvements in skull lengths, tracheal deformities, dentition, motility, spinal stability and eye and nasal secretions were observed [105]. Similar effects were obtained also with subcutaneous weekly administration, which further improved articular cartilage and endurance in motility, and produced a GAG reduction in urine and tissues [106].

#### 6.4.4. Stop Codon Read-Through

For MPS VI patients carrying nonsense mutations, a potential therapeutic approach is represented by pharmacological stop codon read-through, exploiting small molecules able to suppress the effect of premature stop codon termination and restore the production of the full-length protein. The most promising drugs are the aminoglycoside antibiotics gentamicin and PTC124, an orally administrable non-aminoglycoside compound. PTC124 was able to rescue ASB enzyme activity in presence of c.438G > A [p.(Trp146*)], c.943C > T [p.(Arg315*)] and c.979C > T [p.(Arg327*)] variants, while gentamicin was efficacious on c.438G > A [p.(Trp146*)] and c.966G > A [p.(Trp322*)] variants [107,108]. In some cases, also an increased lysosomal localization of ASB enzyme [107] and a reduction of lysosomal size [108] were observed after treatment.

## 7. Experimental Models and Pathogenesis

### 7.1. In Vitro Studies

Since the first description of the ASB deficit in patients’ fibroblasts by Barton and Neufeld in 1972 [3], several in vitro studies on MPS VI pathogenesis have been conducted on different cell types. Lack of enzyme activity was repeatedly shown in MPS VI human skin fibroblasts [63,108,112,113,114,115,116] as well as in other human cells [117]. It was also described in cat fibroblasts [64] and myoblasts [92], in feline leukocytes [118] and in mouse primary osteoclasts [119] of the related MPS VI models. GAG accumulation was commonly described inside lysosomes [63,92,102,119], while in feline leukocytes the ultrastructure of specific granules in both eosinophils and basophils was altered, with the presence of vacuolated cells [118]. Recently, intra- and extracellular CS distribution and its partial co-localization with Golgi and lysosomes was demonstrated [102]. Increased number and size of lysosomes, common markers of lysosomal storage disorders, was evidenced in primary osteoclasts of the MPS VI mouse model, and in human MPS VI fibroblasts [108,119]. Studies conducted in human pulmonary artery endothelial cells, silenced with siRNA showed that in high DS concentration, they displayed reduced viability, reduced expression of endothelial nitric oxide synthase, and an altered expression of natriuretic peptide type C and VEGFA [120].

In human MPS VI fibroblasts, lysosomal ability to recycle metabolites is impaired, as measured by increased ubiquitin levels [121]. These cells showed increased levels of the vescicular autophagic markers LC3II and p62, but with a good colocalization with the lysosomal marker LAMP2, suggesting that autophagosome-lysosome fusion was not completely blocked [121]. However, the same authors observed increased levels of the mitochondrial marker COX IV and a reduction in the mitochondrial membrane potential, suggesting an accumulation of dysfunctional mitochondria due to impaired autophagy [121].

### 7.2. Animal Models

**Figure 4:**
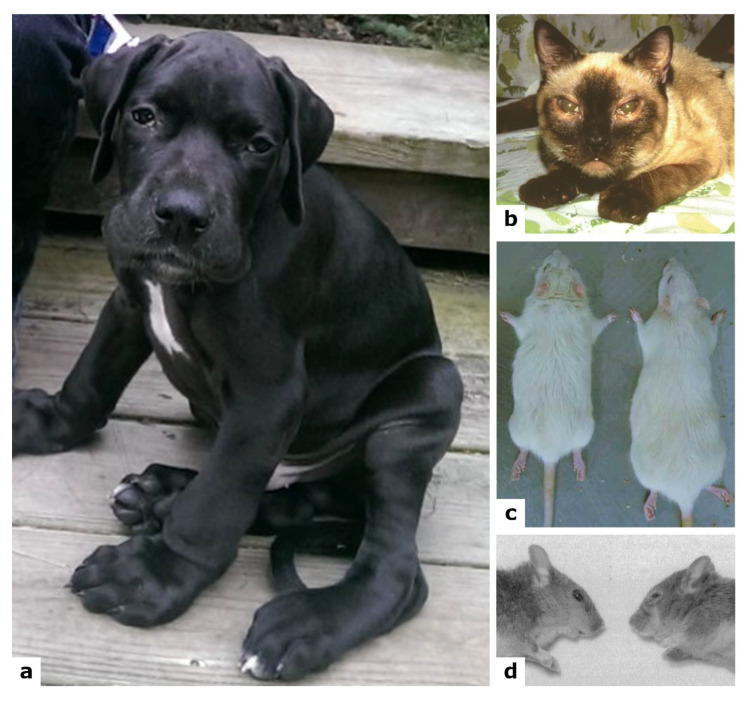
Photographs of some MPS VI animal models. (**a**) Great Dane spontaneous mutant at 4 months of age (reproduced with permission from [124]). (**b**) Siamese cat spontaneous mutant (reproduced with permission from [125]). (**c**) Rat spontaneous model (on the left) compared with wild type (on the right) at 6 months of age (reproduced with permission from [126]). (**d**) Mouse knock-out model (on the right) compared with wild type (on the left) aged 15 months (reproduced with permission from [122]).

Several animal models were described for MPS VI (Figure 4, Table 2), all of them spontaneous except for the mouse models, the first of which were generated in 1996 by a targeted disruption procedure [122] and the second being a descent of a male wild type mouse mutagenized with N-ethyl-N-nitrosourea [123]. Although at the time of the first mouse generation, when both the cat and rat models were available, the small mouse model surely presented important advantages vs. the available larger models, in terms of lifespan and breeding [122]. 

Most models very much resemble the human MPS VI pathology, or at least many aspects of it [125]. Being the only model available for more than 15 years, the cat was the most used animal. Following the description of the other models, experimental procedures were conducted in the different animals according to the experimental aims. All models were extremely useful also for the comprehension of some clinical and molecular signs, which are detailed in the next paragraphs.

#### 7.2.1. Feline Spontaneous Model

An ASB deficient Siamese cat was first identified in 1977 [64]. The cat displayed features reminiscent of the human pathology starting from 6 weeks of age [130], including a broad flattened face, small ears, corneal clouding, large forepaws, pectus excavatum, crouched posture with cervical rigidity, shortened maxilla and progressive reduction in flexibility [65,93,130,131,132] (Figure 4b); moreover, affected animals are smaller than wild type littermates [93,130]. Residual ASB activity (between 1–6%) was found in peripheral blood granulocytes, leukocytes, cultured fibroblasts, liver, kidney, skeletal muscle and pancreas, with consequent accumulation of dermatan-sulphate, detected in urine, myoblasts and chondrocytes [86,93,131,132,133,134,135,136]. A severe, progressive skeletal disease was displayed with bone lesions, significant reduction in bone size, lower bone formation rate and fusion of cervical vertebrae. The mutant cats also showed degeneration of joints, as well as cartilage erosion [65,93,130,131,132,137]. All this leads to progressive locomotor difficulty [65,93]. At a microscopic level, different cell types present membrane-bound cytoplasmic vacuoles, either empty or containing granular material [65,86,130,131,132]. In particular, chondrocytes have an abnormal morphology, increased in size and filled with lysosomal vacuoles [132].

#### 7.2.2. Rat Spontaneous Model

The mutant rat for the *ARSB* gene was detected in 1988 and characterized in 1993 [127], with a small body, short limbs, short and thick tail, and a typical facial dysmorphia, starting from 3 weeks of age [111,127,138,139] (Figure 4c). A reduced ASB activity was observed in several organs, with subsequent accumulation of GAGs [111,121,127,138,140]. At cellular level, a common feature to different cell types is the presence of single membrane vacuoles, containing storage material, except for brain tissue [127,138,139,140]. A severe phenotype was noticed in the cartilage, with an irregular growth plate contributing to the abnormal bone formation and to a reduced motor activity; also, widened, irregular and collapsed tracheas were observed [111,127]. Different inflammatory markers were found altered in serum, cartilage, and synovium, as TNF-α and RANKL [111], as well as elevated levels of COX-2, p38, and TGF-β in MPS fibroblast-like synoviocytes, each of which are downstream mediators of TNF-α [104]. Plasma levels of ceramide and sphingosine-1-phosphate (S1P) were found elevated in MPS VI rat, the first being a proapoptotic lipid contributing to cell death in chondrocytes, and the second being a lipid contributing to the hyperplasia seen in MPS VI synovial tissue [104]. In fact, elevated levels of TUNEL staining were observed in MPS VI rat articular cartilage, a hallmark of apoptosis, together with a decreased expression of the collagens IIA1 and X, all contributing to the impairment of the cartilaginous tissue [104,111,121]. Interestingly, in the rat model, a high number of autophagic vacuoles and increased levels of ubiquitin and of COX IV were observed in visceral organs, showing an altered activation of the autophagic pathway and a disrupted mitophagy [121].

#### 7.2.3. Mouse Models

The first mouse model for MPS VI was generated and characterized in 1996 [122]. The model showed GAG accumulation in urine, liver, kidney, spleen, heart, skin, cornea and optic nerves, starting from 1 month of age [122,141]. Bone disease in this model is very severe, since mice present, starting from 4 weeks of age, the typical facial dysmorphia of the human pathology, with a broadened head and a shortened nose, due to abnormalities of skull bones [122] (Figure 4d). Moreover, they present a shortening of all skeletal elements, due to abnormalities in the growth plate, and an increased thickness of cartilage in trachea, with the presence of vacuolated chondrocytes, also accumulating GAGs [119,122]. At 12 weeks of age, they display a high bone mass with shortened and broadened long bones, ribs exhibiting thickened and wavy irregular surfaces, and pelvic abnormalities [119,122]. Albeit having a normal fertility and life expectancy, they present a reduction in body weight at 9 months of age, compared to age-matched controls [122]. On the opposite of what is commonly observed in patients, no corneal clouding was observed in this model; however, other cornea alterations ranged from a mild disarrangement of fibrils to severe stromal disarray [141]. Lastly, a severe cardiac pathological phenotype with valve thickening was reported for this animal.

A second mouse model was obtained by mutagenesis using N-ethyl-N-nitrosourea [123]. These mutants show a skeletal phenotype consisting of a shortened snout, skull length, maxilla and limbs, wide-set eyes, thicker than normal tail, as well as cartilage thickness in trachea and femoral growth plate. In addition, they present a combination of delayed muscle and nerve degeneration together with a severe hearing loss and corneal abnormalities (opacification, reduction in the corneal epithelium thickness and number of epithelial cell layers, morphological malformations in the stroma). GAG accumulation in all major parenchymal organs, cornea and urine was observed [102,103,123].

#### 7.2.4. Dog Spontaneous Mutants

A first miniature poodle dog, a spontaneous mutant for *ARSB* gene, identified in 2012, showed increased concentration of DS in urine and no ASB activity in fibroblasts [128]. Clinically, the dog presented shortened vertebral bodies, corneal opacities, and heart enlargement with severe thickening of valves. Histologically, metachromatic intracytoplasmic inclusion in neutrophils, leukocytes and monocytes were found, as well as connective tissue lesions with vacuolated fibroblasts. Spleen, smooth muscles of intestines, pancreas and liver showed an accumulation of a large number of macrophages [128].

A miniature schnauzer dog, spontaneous mutant for the *ARSB* gene, was described in 2015, presenting hepatomegaly and incomplete mineralization of the endplates, as well as accumulation of DS in urine. At necropsy, severe gross developmental skeletal abnormalities, diffuse endocardiosis of the mitral valve, marked pancreatomegaly, and moderate cortical thinning of the kidneys were identified. Microscopically, the animal showed swollen hepatocytes with intracytoplasmic vacuoles, which were also identified in renal tubular epithelial cells, chondrocytes of bronchial cartilage, and macrophages in many organs [129].

In 2018, Wang and colleagues identified a third *ARSB* spontaneous mutant dog (Figure 4a). At diagnosis, the Great Dane puppy presented severe generalized skeletal abnormalities, mild corneal opacities and facial dysmorphia. They identified DS accumulation in urine and less than 5% of ASB activity in leukocytes, liver and spleen. Cytoplasmic vacuolation was found in corneal and uveal stroma, heart valves, Kupffer cells and spleen macrophages. Moreover, the trachea and ribs contained severe cytoplasmic vacuolation in chondrocytes and fibroblasts [124].

## 8. Biomarkers

In MPS VI, as well as in all MPSs, GAGs accumulate in lysosomes and in the extracellular matrix and then they are excreted in urine, thus representing a useful, non-invasive diagnostic biomarker. After the advent of ERT, urinary GAG levels were also used to evaluate its efficacy, as they rapidly reduce after initiation of treatment, respond to dosage changes, and reflect restoration of enzyme activity in affected tissues, as also reported in a recent paper [142]. However, this remains an open issue. In a previous report total urinary GAGs were not considered an ideal efficacy biomarker as they do not reflect the disease burden of the affected patients and its improvement after therapy [143].

In MPS VI patients, GAGs have been also evaluated as potential prognostic biomarkers. An association between pre-therapy GAGs and clinical course was first reported in 2005 in a study evaluating 121 MPS VI patients [43]. High urinary GAG values (>200 μg /mg creatinine) were associated with an accelerated clinical course. Conversely, urinary GAG levels below 100 μg/mg creatinine were reported for patients with slowly progressing phenotypes and for most patients over 20 years old, suggesting an association with a longer-term survival [43]. These findings were confirmed in 2014 when a resurvey study analyzed data from the same patients that meanwhile had received ERT for about 10 years [50]. In this paper, authors stated that the pre-therapy urinary GAG levels could be used also to predict ERT efficacy; indeed, patients with urinary GAGs > 200 μg /mg creatinine showed a stabilization with ERT, while the greatest improvements in height and endurance were evidenced mainly in patients with lower baseline urinary GAG levels [50].

Besides urinary total GAGs, single GAG species, as well as oligo- and di-saccharides molecules, have also been evaluated as specific biomarkers for single MPSs. Recently, a simple urinary single assay has been set up for the diagnosis of 10 MPSs. The method measures the oligosaccharides with non-reducing termini specific to each MPSs, thus representing a signature for each disorder [144]. The protocol was also tested in an MPS VI patient that had received ERT for 10 years showing to be potentially useful also in monitoring of therapeutic efficacy [144].

In the last 10 years, also other potential biomarkers, not directly associated to the enzyme deficits, were investigated for MPSs. Heparin cofactor II-thrombin complex (HCII-T) resulted significantly increased in the serum of MPS VI, MPS I and MPS II patients, as well as in the CSF from MPS IH patients [145,146]. It decreased in response to treatment, making it a good candidate biomarker of both disease and therapeutic efficacy [145].

Higher levels of TNF-α compared to healthy controls were detected in MPS patients, including MPS VI, and associated with increased chronical pain and decreased physical abilities [147].

Another key pathological feature of MPSs is the disruption of the extracellular matrix [148]. Batzios and colleagues evidenced in one MPS VI patient increased serum activity of matrix metalloproteinase MMP9 which reduced with ERT until 4 months post-treatment [149].

Another, more recent study evaluated matrix disruption, by quantifying specific collagen breakdown products using liquid chromatography tandem mass spectrometry (LC-MS/MS) method. Results evidenced an altered collagen turnover in MPS patients and more specifically a 3.9-fold increase of glycosylated hydroxylysine Lys-O-GalGlc in five MPS VI patients [150].

In a few studies, also untargeted approaches were applied. A label-free quantitative proteomics approach, followed by a targeted proteomic MRM LC-MS/MS assay was used to identify candidate urinary biomarkers in MPS patients; protein HEG1 was the only marker raised significantly in the MPS VI group, with a 1.9-fold elevation compared to controls [151]. Finally, an integrative untargeted and targeted metabolomics urinary profiling recently evidenced the arginine-proline, histidine and glutathione metabolisms as the main altered pathways in MPS VI patients compared to controls [152]. These pathways are involved in regulating autophagy and protecting from oxidative stress; however, more targeted validation studies are required to explore them in a deeper manner and to possibly highlight new candidate biomarkers.

## 9. Conclusions

Due to the involvement of many organs and systems and its progressive and phenotypically variable nature, Mucopolysaccharidosis type VI is a complex disease that needs a continuous and multi-disciplinary approach to be managed in the best way.

Although enzyme replacement therapy has obtained positive results in several clinical outcomes, some tissues (including bones, cartilage, eye, heart valves and CNS) remain untreated, due to poor vascularization or to the presence of anatomical and physiological barriers. Therefore, thanks to the availability of different animal models of the disease, other possible therapeutic strategies for MPS VI are still actively studied; they include alternative administration routes for ERT, gene therapy, substrate reduction therapy, anti-inflammatory and pharmacological read-through drugs.

In addition, since any therapeutic intervention is more effective when applied in the early phases of the disease, more attention to the very precocious signs, helping to speed up a timely correct diagnosis, would certainly improve the therapeutic outcome. To this aim, it becomes very important to involve and attention different clinical specialists, including orthopedists, cardiologists, and ophthalmologists in the process of diagnosis and in the first phases of the disease. In the same direction, the identification of new biochemical or molecular disease biomarkers would help to obtain an early diagnosis, a possible early prognosis and a reliable monitoring of therapeutic efficacy.

## Figures and Tables

**Table 1:** Experimental therapies evaluated for the treatment of MPS VI. hHSCs = human hematopoietic stem cells, hBM-MSCs = human bone marrow-derived mesenchymal stem cells; hDP-MSCs = human dental pulp-derived mesenchymal stem cells; B4GalT7 = β-1,4-galactosyltransferase.

Therapy	Description	In Vitro Studies		Animal Studies			Clinical Trials		
		Model	Ref	Model	Delivery Route	Ref	Phase	EudraCT Number	Clinical Trials.gov Identifier
Gene therapy	*ARSB* gene transfer mediated by retroviral vectors	feline muscle cells	[92]	cat	intravenous	[93]	-	-	-
	*ARSB* gene transfer mediated by adeno-associated viral vectors	-	-	cat	subretinal	[94]	I/II	2016-002328-10	NCT03173521
				cat	intravenous	[95,96,97]			
				mouse	intravenous	[97,98,99]			
	*ARSB* gene transfer mediated by lentiviral vectors	human fibroblasts, feline chondrocytes	[100]	rat	injection into the knee joint	[100]	-	-	-
		hHSCs, hBM-MSCs, hDP-MSCs	[101]						
Substrate reduction therapy	Odiparcil (substrate analogue for B4GalT7)	human fibroblasts	[102]	mouse	oral	[102,103]	II	2017-002158-35	NCT03370653
Anti-inflammatory drugs	Infliximab (monoclonal antibody against human TNF-α)	-	-	rat	intravenous	[104]	-	-	-
	Pentosan polysulfate	-	-	rat	oral	[105,106]	-	-	-
				rat	subcutaneous	[106]			
Stop codon read-through	Gentamicin	human fibroblasts	[107,108]	-	-	-	-	-	-
	PTC124	human fibroblasts	[107,108]	-	-	-	-	-	-

**Table 2:** Animal models for Mucopolysaccharidosis type VI. ENU = N-ethyl-N-nitrosourea.

Year of Publication	Animal Model	Model Generation	Reference
1977	Siamese cat	Spontaneous	[64]
1993	Rat	Spontaneous	[127]
1996	Mouse	Targeted disruption	[122]
2009	Mouse	ENU mutagenesis	[123]
2012	Miniature Poodle dog	Spontaneous	[128]
2015	Miniature Schnauzer dog	Spontaneous	[129]
2018	Great Dane dog	Spontaneous	[124]

## Data Availability

Not applicable.
